# Heat Shock Protein 90 Triggers Multi-Drug Resistance of Ovarian Cancer via AKT/GSK3β/β-Catenin Signaling

**DOI:** 10.3389/fonc.2021.620907

**Published:** 2021-03-02

**Authors:** Lan Yin, Yuhan Yang, Wanglong Zhu, Yu Xian, Zhengyu Han, Houyi Huang, Liaotian Peng, Kun Zhang, Ye Zhao

**Affiliations:** School of Bioscience and Technology, Chengdu Medical College, Chengdu, China

**Keywords:** multi-drug resistance, heat shock protein 90, ovarian cancer, β-catenin, chemotherapy

## Abstract

Ovarian cancer is the most lethal gynaecologic tumor, with which multi-drug resistance as the major therapeutic hindrance. Heat shock protein 90 (Hsp90) has been involved in cancer malignant behaviors. However, its role and mechanism in multi-drug resistance of ovarian cancer remains poorly understood. Our results demonstrated that Hsp90 was overexpressed in multi-drug resistant ovarian cancer cells. Hsp90 downregulation by shHsp90 or inhibitor BIIB021 increased the sensitivity of multi-drug resistant ovarian cancer cells to paclitaxel and cisplatin, and augmented the drugs-induced apoptosis. Hsp90 positively regulated the expressions of multi-drug resistance protein 1 (P-gp/MDR1), breast cancer resistance protein (BCRP), Survivin and Bcl-2 expressions closely associated with multi-drug resistance. Moreover, overexpression of Hsp90 promoted β-catenin accumulation, while Hsp90 downregulation decreased the accumulation, nuclear translocation and transcriptional activity of β-catenin. We also identified that β-catenin was responsible for Hsp90-mediated expressions of P-gp, BCRP, Survivin, and Bcl-2. Furthermore, Hsp90 enhanced the AKT/GSK3β signaling, and AKT signaling played a critical role in Hsp90-induced accumulation and transcriptional activity of β-catenin, as well as multi-drug resistance to paclitaxel and cisplatin. In conclusion, Hsp90 enhanced the AKT/GSK3β/β-catenin signaling to induce multi-drug resistance of ovarian cancer. Suppressing Hsp90 chemosensitized multi-drug resistant ovarian cancer cells via impairing the AKT/GSK3β/β-catenin signaling, providing a promising therapeutic strategy for a successful treatment of ovarian cancer.

## Introduction

Ovarian cancer is one of the most prevalent female reproductive organ malignancies and the major cause of gynecological malignancy-related mortality (1), primarily treated with surgery and chemotherapy (2, 3). Although ovarian cancer treatment has growingly advanced over the recent decades, the 5-year survival has slowly increased (4–6). Paclitaxel- and platinum-based therapy have been long recommended as the cornerstone for the primary management of ovarian cancer (7–9). However, the occurrence of multi-drug resistance (MDR) in ovarian cancer has greatly limited therapeutic efficiency of anti-cancer drugs including paclitaxel, carboplatin and cisplatin, ultimately leading to treatment failure (10–12). Therefore, it is urgent to understand the mechanism involved and thus overcome MDR for successful therapy of ovarian cancer.

MDR in cancer cells is a multi-factor, multi-stage process that varies between different drugs and tumor types (13, 14). The potential mechanisms of MDR include ectopic activation of adenosine triphosphate (ATP)-binding cassette (ABC) transporter family and anti-apoptosis induction (15, 16). Cancer patients who are resistant to chemotherapy often exhibit high expression of various ABC transporter efflux pumps including, multi-drug resistance protein 1 (P-gp/MDR1), MDR-associated protein 1 and breast cancer resistance protein (BCRP) (17, 18), the ABC transporter family members requiring ATP hydrolysis to efflux substrates and cytotoxic substances from cells (19). Increasing studies have revealed that ABC transporter can be used as a target to reverse MDR (20). Moreover, abnormal expression of anti-apoptotic protein is also closely correlated with chemoresistance (21). Previous studies revealed that Survivin and Bcl-2 upregulation can suppress the anti-cancer drug-induced apoptosis in a series of cancers, such as ovarian, breast, and lung cancer (22–24). Cancer cells undergoing MDR were generally characterized by ectopic alteration of various pathways including PI3K/AKT and Wnt/β-catenin, which play an import role in the expression of ABC transporter and anti-apoptosis protein such as P-gp, BCRP, Survivin and Bcl-2 (25–30).

Heat shock proteins (HSPs) are molecular chaperones that promote cell survival in response to environmental stress conditions (31). As a member of HSPs, 90KD heat-shock protein (Hsp90) interacts with over 200 client proteins involved in several cellular functions and signaling pathways, as well as has factored into cell proliferation, differentiation, and apoptosis (32–35). Accumulating evidences have shown that Hsp90 is overexpressed in multiple tumors including cervical, and breast cancer and osteosarcoma, and is implicated in cancer malignant behaviors such as invasion, distant metastasis and immune escape (36, 37). Cancer cells with sustained high expression of Hsp90 also exhibit resistance to chemotherapy (38). Inhibition of Hsp90 enhances certain anti-cancer drug-induced apoptosis in aggressive cancer (39, 40). So far, Hsp90 has been one of the most extensively investigated targets for cancer therapy (41). The synthetic small-molecular inhibitor targeting ATP-binding pocket of Hsp90, such as BIIBO21, is usually employed to improve the chemotherapeutic efficacy of tumor cells (42). However, its mechanism of Hsp90 in MDR of ovarian cancer remains to be completely clarified. Therefore, this study intended to explore the role of Hsp90 in mediating the MDR of ovarian cancer cells to paclitaxel and cisplatin as well as the underlying mechanism.

## Methods

### Reagents and Antibodies

Paclitaxel (Taxol), cisplatin (CDDP), dimethyl sulfoxide (DMSO), 3-(4,5-Dimethylthiazol-2-yl)-2,5-diphenyl-tetrazolium bromide (MTT), were obtained from Sigma-Aldrich (St Louis, MO, USA). The culture medium RPMI-1640, penicillin, and streptomycin were purchased from HyClone Laboratories (Logan, UT, USA). Fetal bovine serum (FBS) was purchased from Gibco (Waltham, CA, USA). The compound BIIB021 (Hsp90 inhibitor), Tariquidar (P-gp inhibitor) and Ko143 (BCRP inhibitor) were purchased from Selleck Chemicals (Houston, TX, USA). shHsp90 (target sequence, 5'-TCCACGAAGACTCCACTAA-3′), shβ-catenin (target sequence, 5′-ATGCACAAGAATGGATCACAA-3′), shAKT (target sequence, 5′-GCTTCTATGGCGCTGAGATTG-3′), and shRNA negative control (shNC) were gained from GenePharma (Shanghai, China). cDNAs encoding Hsp90 were cloned into pcDNA3.1 to generate the Hsp90 expression vector pcDNA3.1-Hsp90. pcDNA3.0-D88N-Hsp90 was a gift from William Sessa (43). Lipofectamine 3000 transfection reagent was obtained from Invitrogen (Carlsbad, CA, USA). BCA protein assay kit, Radioimmunoprecipitation (RIPA) lysis buffer and Nuclear and Cytoplasmic Protein Extraction kit were purchased from Beyotime Biotechnology (Nantong, Jiangsu, China). Primary antibodies against β-catenin, Lamin B1 and GAPDH were obtained from Santa Cruz Biotechnology (Santa Cruz, CA, USA). Primary antibodies against P-gp, BCRP, Hsp90, Bcl-2, Survivin, AKT, phospho-AKT(Ser473), glycogen synthase kinase (GSK3β), phospho-GSK3β (Ser9), and non-phospho (active) β-Catenin (unphosphorylated by GSK3 at Ser33/37/Thr41) were obtained from Cell Signaling Technology (Danvers, MA, USA). The hsorseradish peroxidase (HRP)-conjugated secondary antibody was purchased from ZSGB-bio (Peking, China).

### Cell Culture

The human ovarian-originated cancer cell line A2780 and its paclitaxel- and cisplatin-resistant sublines (A2780/Taxol and A2780/CDDP cells) were obtained from KeyGEN Biotech Co. (Nanjing, Jiangsu, China). Cells were authenticated by STR profiling, cultured in RPMI-1640 supplemented with 1% penicillin/streptomycin sulfate, and 10% FBS, and maintained at 37°C in a humidified 5% CO_2_ incubator. The drug-resistance phenotype of A2780/Taxol cells or A2780/CDDP cells was maintained by culturing the cells in a growth medium containing 0.3 μM paclitaxel or 1 μM cisplatin. When cells reached 80% to 90% confluency, they were incubated with 0.25% Trypsin and then passaged.

### Cell Transient Transfection

Cells were seeded in 96- or 6-well-plates and incubated overnight. The confluent cells (70%−90%) were transfected with shNC, shHsp90, shβ-catenin, pcDNA3.1/pcDNA3.0, pcDNA3.1-Hsp90, or pcDNA3.0-D88N-Hsp90, or pcDNA3.0-AKT using Lipofectamine 3000 transfection reagent following the manufacturer's instructions. Seventy-two hours after transfection, cells were collected and analyzed by the MTT assay, flow cytometry analysis (FACS), or western blotting.

### MTT Assay

MTT assays were performed out to examine drug cytotoxicity. Briefly, cells (5 × 10^3^/well) were seeded in a 96-well-plate with 100 μl RPMI 1640 and 5% FBS for 24 h. Cells were treated with 1 μM BIIB021 or transfected with shHsp90, or pcDNA3.1-Hsp90 for 24 h then cultured in medium with various concentrations of paclitaxel (Taxol, 0.1, 0.2, 0.4, 0.8, 1.6 μM) or cisplatin (CDDP, 1, 2, 4, 8, 16 μM) for an additional 48 h. MTT dye solution was added to each well at final concentration of 0.5 mg/mL and incubated for 4 h at 37°C. The medium was discarded, and 150 μL of DMSO was added into each well to stop the reaction. Cell viability was evaluated by measuring the absorbance at 490 nm in an Ultra Microplate Reader (Bio-Tek Instruments, Winooski, VT, USA). Paclitaxel and cisplatin concentrations that achieved 50% growth inhibition (IC50) were calculated from survival curves using the Bliss method.

### Western Blotting

Protein lysates were obtained using RIPA lysis buffer for western blotting. The cytoplasmic and nuclear proteins were extracted using Cytoplasmic Protein Extraction kit. Protein concentration was assessed using BCA protein assay kit. Equal quantities of proteins (40 μg/sample) were loaded in each lane on sodium dodecyl sulfate-polyacrylamide gels (10%) and electrophoresed under reduced conditions. The proteins were then transferred onto polyvinylidene difluoride membranes. Following blocking in 5% skim milk in phosphate buffer solution (PBS) overnight at 4°C, and the membranes were incubated for 2 h at room temperature with primary antibodies prepared in blocking buffer. The membranes were washed 3 times with phosphate buffer solution (PBS) and incubated for 2 h at room temperature with HRP-conjugated secondary antibodies. The membranes were washed 3 times and bands were visualized with an enhanced chemiluminescence detection kit from Invitrogen (Carlsbad, CA, USA) and a Bio-Rad Molecular Imager. A mouse monoclonal anti-GAPDH antibody was used as the control for each sample.

### Flow Cytometry Analysis (FACS)

FACS was performed to analyze cell apoptosis. Briefly, cells were seeded in RPMI 1640 with 5% FBS in a 12-well-plate (2 × 10^5^ cells/well), and then treated with BIIB021 (1 μM) or transfected with shHsp90 for 24 h, followed by treatment with paclitaxel (0.4 μM) or cisplatin (2 μM) for additional 48 h. Adherent cells were detached from the culture plate. Cells (10^6^ cells/mL) were then incubated with Annexin V and Propidium Iodide for 15 min at 4°C and analyzed using a flow cytometer (BD Bioscience; San Jose, CA, USA).

### Immunofluorescence Staining

Cells were seeded on confocal dishes and cultured until they reach 40–60% confluence. Cells were then fixed with 4% paraformaldehyde for 20 min, permeabilized using 0.2% Triton X-100 for 20 min. Permeabilized cells were blocked with 10% goat serum for 2 h at room temperature. The confocal dishes were then incubated with primary mouse monoclonal anti-β-catenin antibodies (1:50) in blocking buffer overnight at 4°C, and subsequently incubated with Alexa Fluor 488 secondary antibodies (1:100) in blocking buffer for 2 h at room temperature. Cells were then counterstained with 5 mg/mL DAPI for 10 min, and subjected to Zeiss confocal microscope.

### Dual-Luciferase Reporter Assay

TOPflash and FOPflash luciferase reporters (Upstate Biotechnology, Lake Placid, NY, USA) are usually used to assay β-catenin transcriptional activity. TOPflash contains SIX wildtype β-catenin/TCF-binding sites upstream of a luciferase reporter gene, while FOPflash contains SIX mutated β-catenin/TCF-binding sites (44). FOPflash is applied as a specific control for TOPflash activity. Cells were seeded in 24-well-plates until 70–90% confluency, and then pcDNA3.1, pcDNA3.1-Hsp90, shNC, shAKT, or shHsp90 was co-transfected with 0.2 μg of TOPflash plus 10 ng of pRL-SV40 or 0.2 μg of FOPflash plus 10 ng of pRL-SV40 using Lipofectamine 3000, as indicated. After 48 h, the TOPflash and FOPflash luciferase activity were detected using a dual-luciferase reporter system (Promega, Madison, WI, USA). The luciferase activity of each sample was normalized against Renilla reporter pRL-SV40 (Promega, Madison, WI, USA) luciferase activity for monitoring transfection efficiency.

### Statistical Analysis

Results from at least 3 independent experiments were expressed as the mean ± standard deviation (SD). Statistical significance was evaluated using a two-tailed *t*-test for comparisons between 2 groups. A one-way ANOVA was used to assess the differences in means between groups. All analyzes were performed using GraphPad Prism Software Version 5.0 (GraphPad Software Inc., La Jolla, CA, USA). A value of *P* < 0.05 was considered statistically significant.

## Results

### Inhibition of Hsp90 Improved the Chemosensitivity of Multi-Drug Resistant Ovarian Cancer Cells to Paclitaxel and Cisplatin

**Figure 1:**
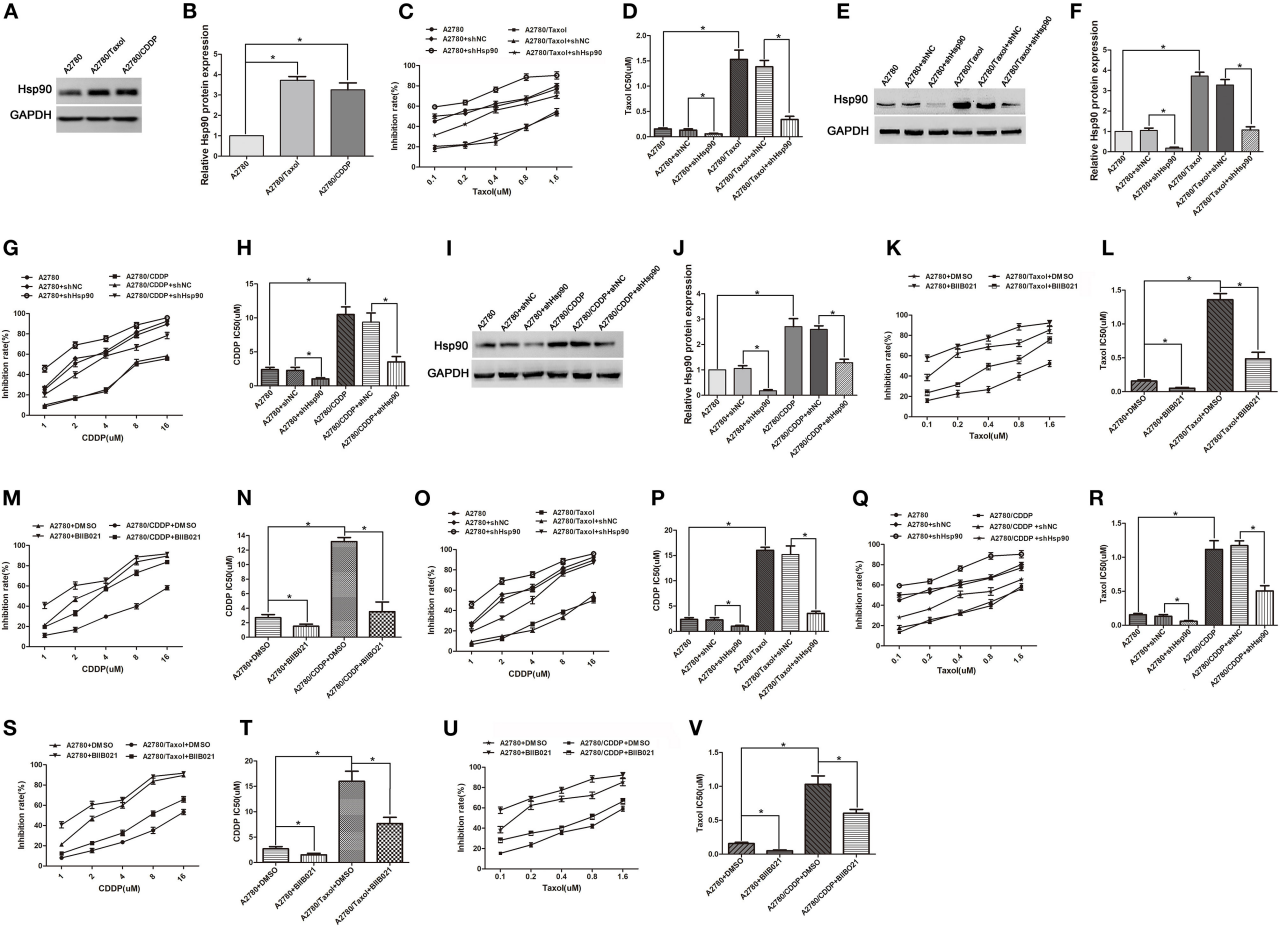
The role of heat shock protein 90 (Hsp90) in multi-drug resistance (MDR) of ovarian cancer. **(A)** Western blotting for Hsp90 expression in A2780, A2780/Taxol, and A2780/CDDP cells. GAPDH was used as the internal control. **(B)** Western blotting to show relative quantitation of Hsp90 expression normalized to GAPDH using Image J analysis. **(C)** The sensitivity of paclitaxel in A2780 and A2780/Taxol cells transfected with/without shNC or shHsp90, was assessed using MTT assays. **(D)** The IC50 of paclitaxel in A2780 and A2780/Taxol cells transfected with/without shNC or shHsp90. **(E,F)** Respective western blots and relative quantitation of Hsp90 in A2780 and A2780/Taxol cells transfected with/without shNC or shHsp90. **(G,H)** Respective sensitivity and IC50 of cisplatin in A2780 and A2780/CDDP cells transfected with/without shNC or shHsp90. **(I,J)** Respective western blots and relative quantitation of Hsp90 in A2780 and A2780/CDDP cells transfected with/without shNC or shHsp90. **(K,L)** Respective sensitivity and IC50 of paclitaxel in A2780 and A2780/Taxol cells treated with DMSO or BIIB021. **(M,N)** Respective sensitivity and IC50 of cisplatin in A2780 and A2780/CDDP cells treated with DMSO or BIIB021. **(O,P)** Respective sensitivity and IC50 of cisplatin in A2780 and A2780/Taxol cells transfected with/without shNC or shHsp90. **(Q,R)** Respective sensitivity and IC50 of paclitaxel in A2780 and A2780/CDDP cells transfected with/without shNC or shHsp90. **(S,T)** Respective sensitivity and IC50 of cisplatin in A2780 and A2780/Taxol cells treated with DMSO or BIIB021. **(U,V)** Respective sensitivity and IC50 of paclitaxel in A2780 and A2780/CDDP cells treated with DMSO or BIIB021. The results of western blotting were analyzed using Image J. The IC50 was calculated from the survival curves generated using the Bliss method (mean ± SD of 3 independent experiments). *P < 0.05.

To examine whether Hsp90 is involved in ovarian cancer resistance, paclitaxel- and cisplatin-resistant ovarian cancer cells (A2780/Taxol and A2780/CDDP), and their parental cells A2780 were used in this study. The western blotting results showed that the protein expression of Hsp90 was significantly increased in A2780/Taxol and A2780/CDDP cells, compared with A2780 cells (Figures 1A,B), suggesting that Hsp90 was related to the resistance of ovarian cancer cells to paclitaxel and cisplatin. To confirm these results, Hsp90 was silenced, and then an MTT assay was conducted to test the inhibition rate of paclitaxel and cisplatin. The results showed that A2780/Taxol and A2780/CDDP cells were, respectively, more resistant to paclitaxel and cisplatin, compared with A2780 cells (Taxol IC50 1.53 μM vs. 0.15 μM; CDDP IC50 10.50 μM vs. 2.38 μM) (Figures 1C–J). After silencing Hsp90, the IC50 of paclitaxel in A2780 and A2780/Taxol cells (0.13 μM vs. 0.06 μM; 1.38 μM vs. 0.34 μM) (Figures 1C–F), and the IC50 of cisplatin in A2780 and A2780/CDDP cells were significantly decreased (2.27 μM vs. 1.02 μM; 9.38 μM vs. 3.52 μM) (Figures 1G–J). Next, the Hsp90 inhibitor BIIB021, was used to further examine the role of Hsp90 in regulating the sensitivity of ovarian cancer cells to paclitaxel and cisplatin. The results showed that BIIB021 significantly reduced the IC50 of paclitaxel in A2780 and A2780/Taxol cells (0.16 μM vs. 0.05 μM; 1.36 μM vs. 0.49 μM) (Figures 1K,L), and the IC50 of cisplatin in A2780 and A2780/CDDP cells (2.69 μM vs. 1.51 μM; 13.17 μM vs. 3.51 μM) (Figures 1M,N). These results suggested that Hsp90 was implicated in the resistance of ovarian cancer cells to paclitaxel and cisplatin.

Furthermore, we determined whether Hsp90 was associated with MDR in ovarian cancer. The results showed that A2780/Taxol and A2780/CDDP cells were also, respectively, more resistant to cisplatin and paclitaxel, compared with A2780 cells (CDDP IC50 16.03 μM vs. 2.38 μM; Taxol IC50 1.12 μM vs. 0.15 μM) (Figures 1O–R), suggesting that A2780/Taxol and A2780/CDDP cells exhibit MDR. The IC50 of cisplatin in A2780/Taxol cells (15.22 μM vs. 3.56 μM) (Figures 1O,P), and the IC50 of paclitaxel in A2780/CDDP cells were significantly decreased (1.17 μM vs. 0.51 μM) (Figures 1Q,R) when Hsp90 was silenced. In addition, the IC50 of cisplatin and paclitaxel, respectively, in A2780/Taxol and A2780/CDDP cells incubated with BIIB021 were significantly reduced (CDDP 15.99 μM vs. 7.65 μM; Taxol 1.03 μM vs. 0.61 μM) (Figures 1S–V). Together, the above results suggested that Hsp90 contributed to MDR, inhibiting Hsp90 re-sensitized multi-drug resistant ovarian cancer cells to paclitaxel and cisplatin.

### The Role of ABC Transports in Hsp90-Mediated Resistance

**Figure 2:**
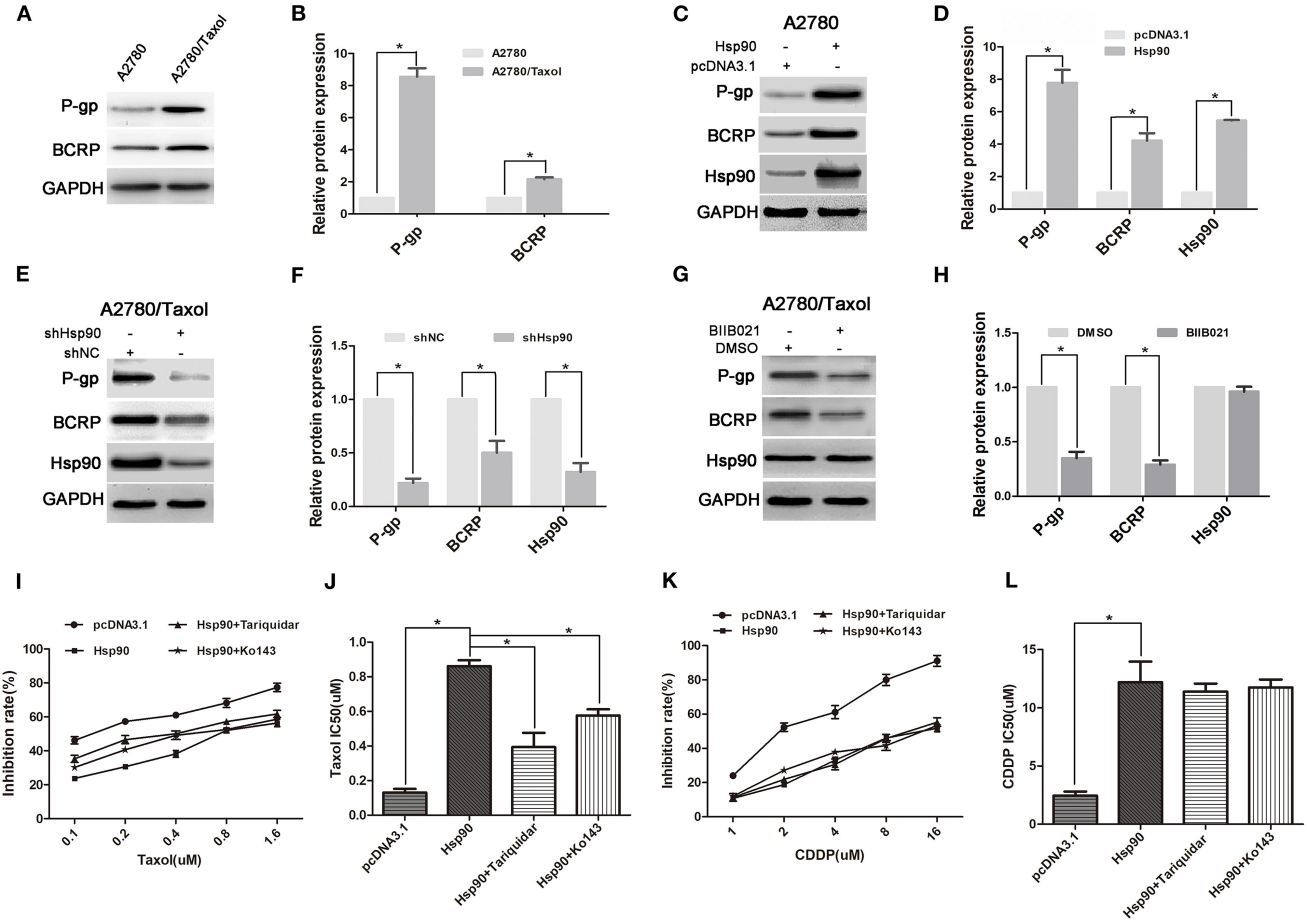
The role of multi-drug resistance protein 1 (P-gp) and breast cancer resistance protein (BCRP) in Heat shock protein 90 (Hsp90) mediated drug-resistance. Respective western blots and relative quantitation of P-gp and BCRP in A2780 and A2780/Taxol cells **(A,B)**, A2780 cells transfected with pcDNA3.1 or pcDNA3.1-Hsp90 for 72 h **(C,D)**, A2780/Taxol cells transfected with shNC or shHsp90 for 72 h **(E,F)**, and A2780/Taxol cells treated with DMSO or 1 μM BIIB021 for 72 h **(G,H)**. Respective sensitivity and IC50 of paclitaxel **(I,J)** and cisplatin **(K,L)** in A2780 cells transfected with pcDNA3.1 or pcDNA3.1-Hsp90 for 24 h followed by treatment with 50 nM Tariquidar or 1 μM Ko143 for additional 48 h. The results of western blotting were analyzed using Image J. The IC50 was calculated from the survival curves generated using the Bliss method (mean ± SD of 3 independent experiments). *P < 0.05.

Ectopic expression of ABC transports is regarded as the main reason of MDR (20, 45). To explore the mechanism of Hsp90 in MDR in ovarian cancer, the expressions of P-gp and BCRP were determined. Compared with A2780 cells, the P-gp and BCRP expression levels were significantly increased in A2780/Taxol cells (Figures 2A,B). Moreover, the pcDNA3.1-Hsp90 expression vector was transfected into A2780 cells and a human normal ovarian surface epithelia cell line (IOSE80). Western blotting results showed that the protein levels of P-gp and BCRP were upregulated by the ectopic expression of Hsp90 in A2780 (Figures 2C,D) and IOSE80 cells (Supplementary Figure 1), while these protein levels were decreased when Hsp90 was silenced using shRNA in A2780/Taxol cells (Figures 2E,F). Similar results were also observed in A2780/Taxol cells treated with BIIB021 (Figures 2G,H). These results indicated that Hsp90 enhanced the expressions of P-gp and BCRP. Furthermore, as shown in Figures 2I–L, the sensitivity of A2780 cells to paclitaxel (IC50 0.13 μM vs. 0.86 μM) was decreased by the ectopic expression of Hsp90, while this decrease was rescued by the P-gp inhibitor Tariquidar (IC50 0.86 μM vs. 0.39 μM) or BCRP inhibitor KO143 (IC50 0.86 μM vs. 0.58 μM). The sensitivity of A2780 cells to cisplatin (IC50 2.46 μM vs. 12.19 μM) was also decreased by the ectopic expression of Hsp90, whereas this reduction cannot be reversed by Tariquidar or KO143. Above results indicated that ABC transports were involved in Hsp90-induced resistance of ovarian cancer to paclitaxel.

### Hsp90 Promoted the Expression of Anti-apoptosis Proteins

**Figure 3:**
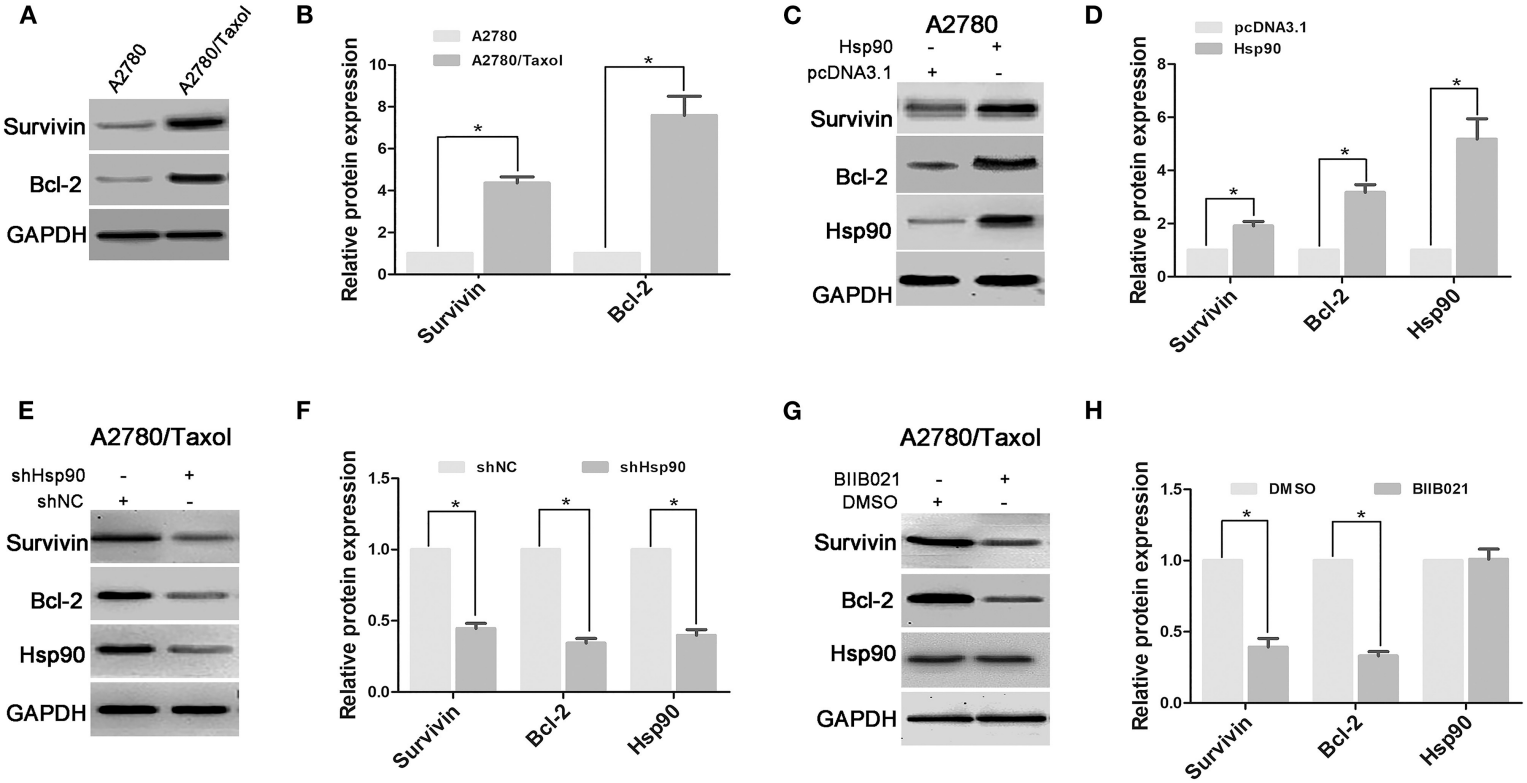
The effect of heat shock protein 90 (Hsp90) on the expressions of Survivin and Bcl-2. Respective western blots and relative quantitation of Survivin and Bcl-2 in A2780 and A2780/Taxol cells **(A,B)**, A2780 cells transfected with pcDNA3.1 or pcDNA3.1-Hsp90 for 72 h **(C,D)**, A2780/Taxol cells transfected with shNC or shHsp90 for 72 h **(E,F)**, and A2780/Taxol cells treated with DMSO or 1 μM BIIB021 for 72 h **(G,H)**. GAPDH was used as the internal control. The results of western blotting were analyzed using ImageJ (mean ± SD of 3 independent experiments). *P < 0.05.

Anti-apoptosis is another crucial mechanism for chemoresistance in cancer cells (21). In this study, the expression levels of anti-apoptosis proteins Survivin and Bcl-2 were measured in A2780 and A2780/Taxol cells. The results showed that Survivin and Bcl-2 expressions were increased in A2780/Taxol cells compared with A2780 cells (Figures 3A,B). Moreover, when Hsp90 was overexpressed in A2780 cells, Survivin and Bcl-2 expressions were enhanced compared with control (Figures 3C,D). Further, Survivin and Bcl-2 in A2780/Taxol cells were downregulated when Hsp90 was silenced using shRNA (Figures 3E,F) or inhibited with BIIB021 (Figures 3G,H). These results suggested that Hsp90 induced Survivin and Bcl-2 expression and was associated with anti-apoptotic mechanisms in multi-drug resistant ovarian cancer cells.

### Inhibition of Hsp90 Enhanced Paclitaxel- and Cisplatin-Induced Apoptosis in Ovarian Cancer Cells

**Figure 4:**
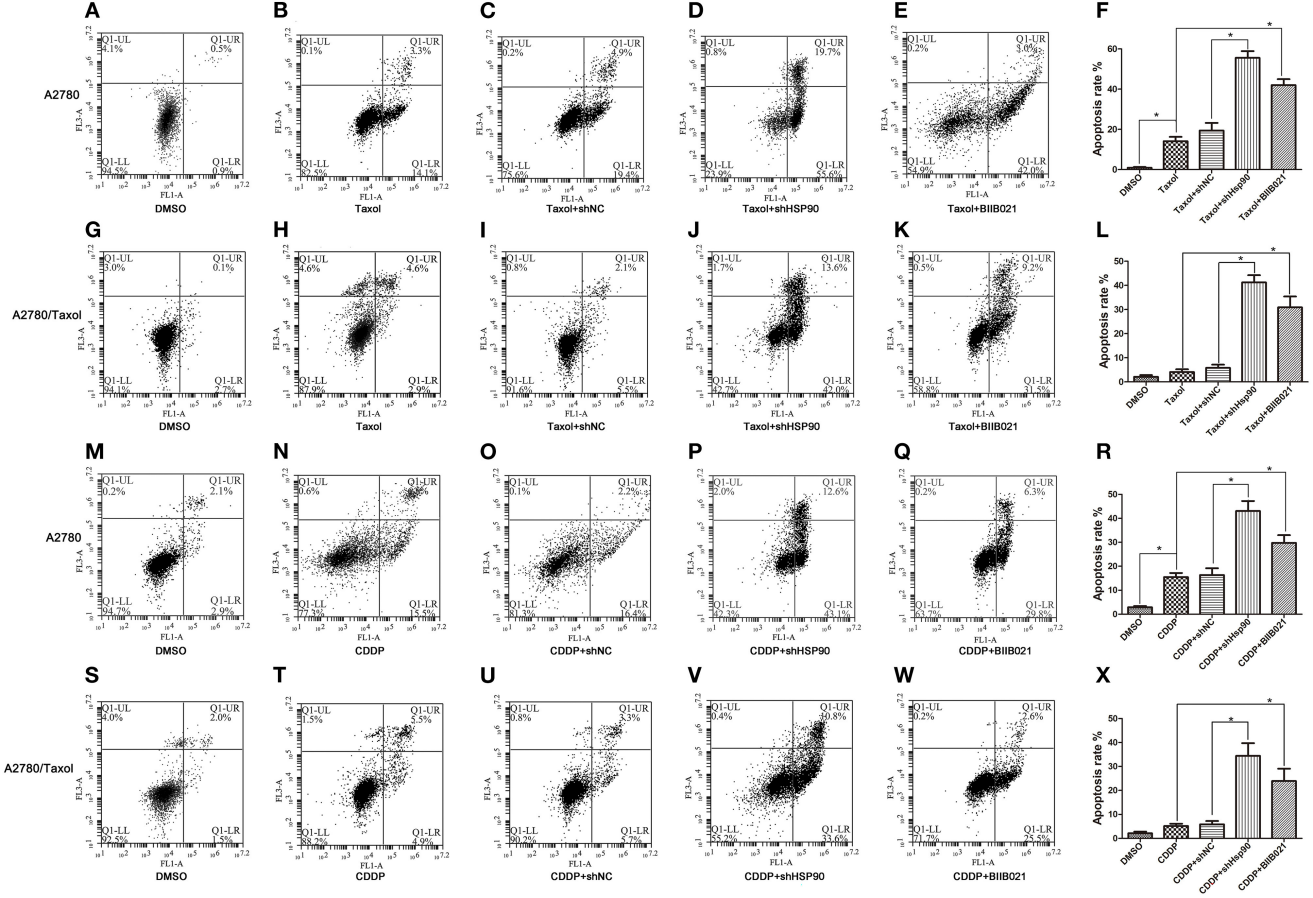
The effect of heat shock protein 90 (Hsp90) on drug-induced apoptosis. Flow cytometry analysis and apoptotic rates in A2780 and A2780/Taxol cells transfected with shNC or shHsp90 for 24 h, or incubated with 1 μM BIIB021 for 24 h, followed by treatment with 0.4 μM paclitaxel **(A–L)** or 2 μM cisplatin for 48 h **(M–X)**, as indicated. Data are expressed as mean ± SD of 3 for each experimental group. *P < 0.05.

To confirm the role of Hsp90 in drug-induced apoptosis, A2780/Taxol cells characterized by MDR and A2780 cells were incubated with paclitaxel or cisplatin, and analyzed by FACS. As shown in Figures 4A–L, the results showed that paclitaxel-induced apoptosis was significantly increased in A2780 and A2780/Taxol cells transfected with shHsp90 (19.4 vs. 55.6%, Figures 4C,D; 5.5 vs. 42.0%, Figures 4I,J) or treated with BIIB021 (14.1 vs. 42.0%, Figures 4B,E; 2.9 vs. 31.5%, Figures 4H,K), as compared with the control. Moreover, the same trend was observed during cisplatin-induced apoptosis. As shown in Figures 4M–X, cisplatin-induced apoptosis was significantly promoted in A2780 and A2780/Taxol cells transfected shHsp90 (16.4 vs. 43.1%, Figures 4O,P; 5.7 vs. 33.6%, Figures 4U,V) or treated with BIIB021 (15.5 vs. 29.8%, Figures 4N,Q; 4.9 vs. 22.5%, Figures 4T,W), as compared with the control. Furthermore, cisplatin- and paclitaxel-induced apoptosis of A2780/CDDP cells were also significantly enhanced by shHsp90 or BIIB021 (Supplementary Figure 3). These results suggested that the inhibition of Hsp90 significantly increased paclitaxel- and cisplatin-induced apoptosis in ovarian cancer cells.

### β-Catenin Was Responsible for Hsp90-Induced Expression of ABC Transports and Anti-apoptosis Proteins

**Figure 5:**
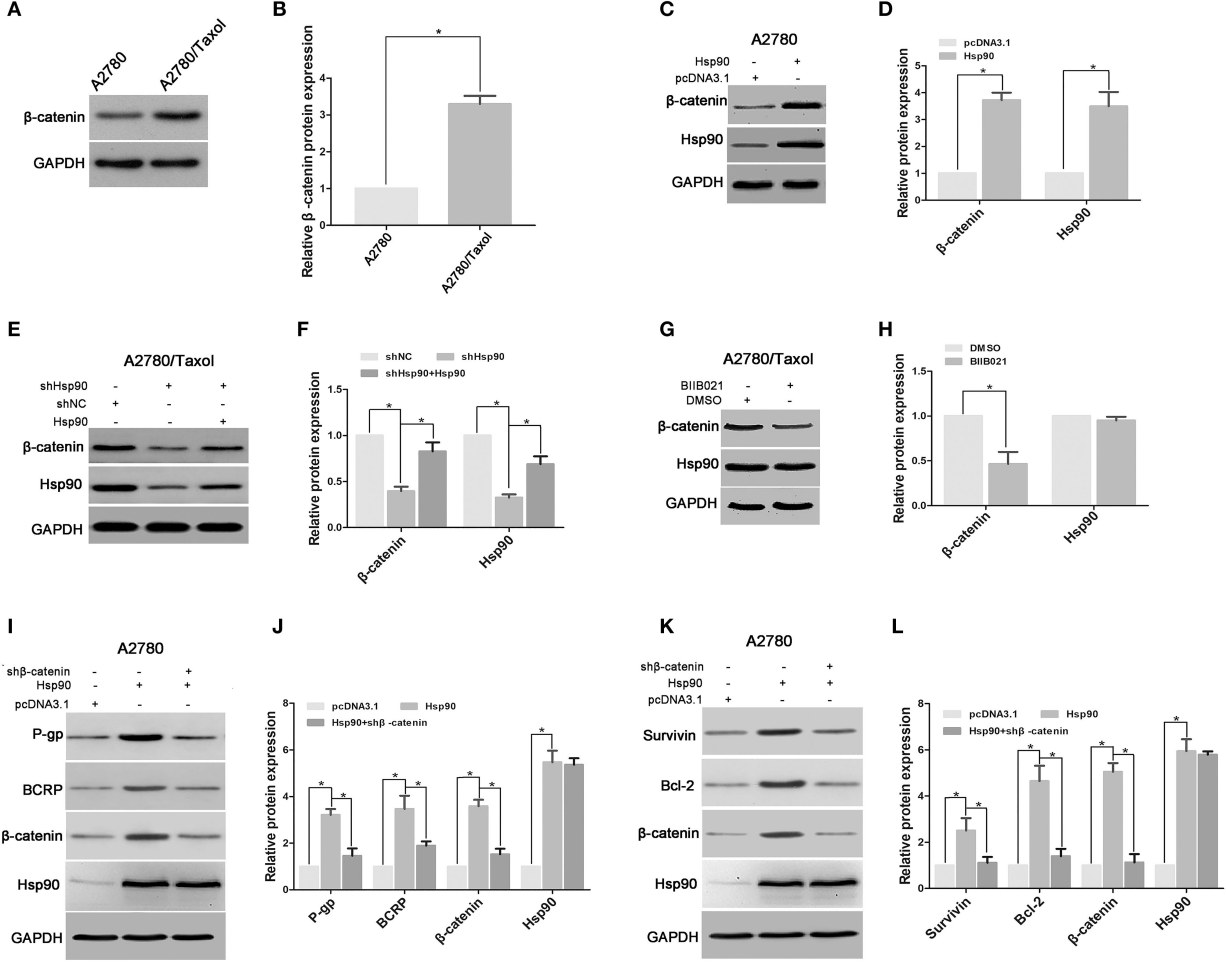
Heat shock protein 90 (Hsp90) increased the expressions of multidrug resistance protein 1 (P-gp), breast cancer resistance protein (BCRP), Survivin and Bcl-2 via β-catenin. Respective western blots and relative quantitation of β-catenin in A2780 and A2780/Taxol cells **(A,B)**. Respective western blots and relative quantitation of β-catenin and Hsp90 in A2780 cells transfected with pcDNA 3.1 or pcDNA 3.1-Hsp90 **(C,D)**, A2780/Taxol cells transfected with shNC, shHsp90, or shHsp90 plus pcDNA 3.1-Hsp90 for 72 h **(E,F)**, A2780/Taxol cells treated with DMSO or 1 μM BIIB021 for 72 h **(G,H)**. Respective western blots and relative quantitation of P-gp, BCRP, β-catenin, and Hsp90 in A2780 cells transfected with pcDNA3.1, pcDNA3.1-Hsp90, or pcDNA3.1-Hsp90 plus shβ-catenin for 72 h **(I,J)**. Respective western blots and relative quantitation of Survivin, Bcl-2, β-catenin, and Hsp90 in A2780 cells transfected with pcDNA3.1, pcDNA3.1-Hsp90, or pcDNA3.1-Hsp90 plus shβ-catenin for 72 h **(K,L)**. GAPDH was used as the internal control, and the results of western blotting were analyzed using ImageJ (mean ± SD of 3 independent experiments). *P < 0.05.

The Wnt/β-catenin signaling pathway is a key regulator of the expression of certain ABC transport and anti-apoptosis proteins (25, 27, 28, 30), so, we next explored the role of β-catenin in the Hsp90-mediated expression of P-gp, BCRP, Survivin, and Bcl-2. The result showed that the protein level of β-catenin was increased in A2780/Taxol cells, compared with A2780 cells (Figures 5A,B). Following ectopic expression of Hsp90 in A2780 cells, western blotting results showed that β-catenin expression was significantly increased as Hsp90 levels increased (Figures 5C,D). A2780/Taxol cells in which Hsp90 was silenced showed the reduction of β-catenin expression compared with control, and this downregulation was rescued by Hsp90 overexpression (Figures 5E,F). The downregulation of β-catenin was also confirmed in A2780/Taxol cells treated with BIIB021 (Figures 5G,H) or transfected with D88N-Hsp90 (Supplementary Figure 2), a dominant negative gene of Hsp90 (43). Moreover, the recombinant vector of Hsp90 was co-transfected with shβ-catenin into A2780 cells. The results showed that Hsp90-induced expression of P-gp, BCRP, Survivin and Bcl-2 was abolished by silencing β-catenin (Figures 5I–L). The above results suggested that Hsp90 enhanced β-catenin expression to upregulate P-gp, BCRP, Survivin and Bcl-2.

### Hsp90 Triggered Cytoplasmic Accumulation, Nuclear Translocation and Transcriptional Activity of β-Catenin

**Figure 6:**
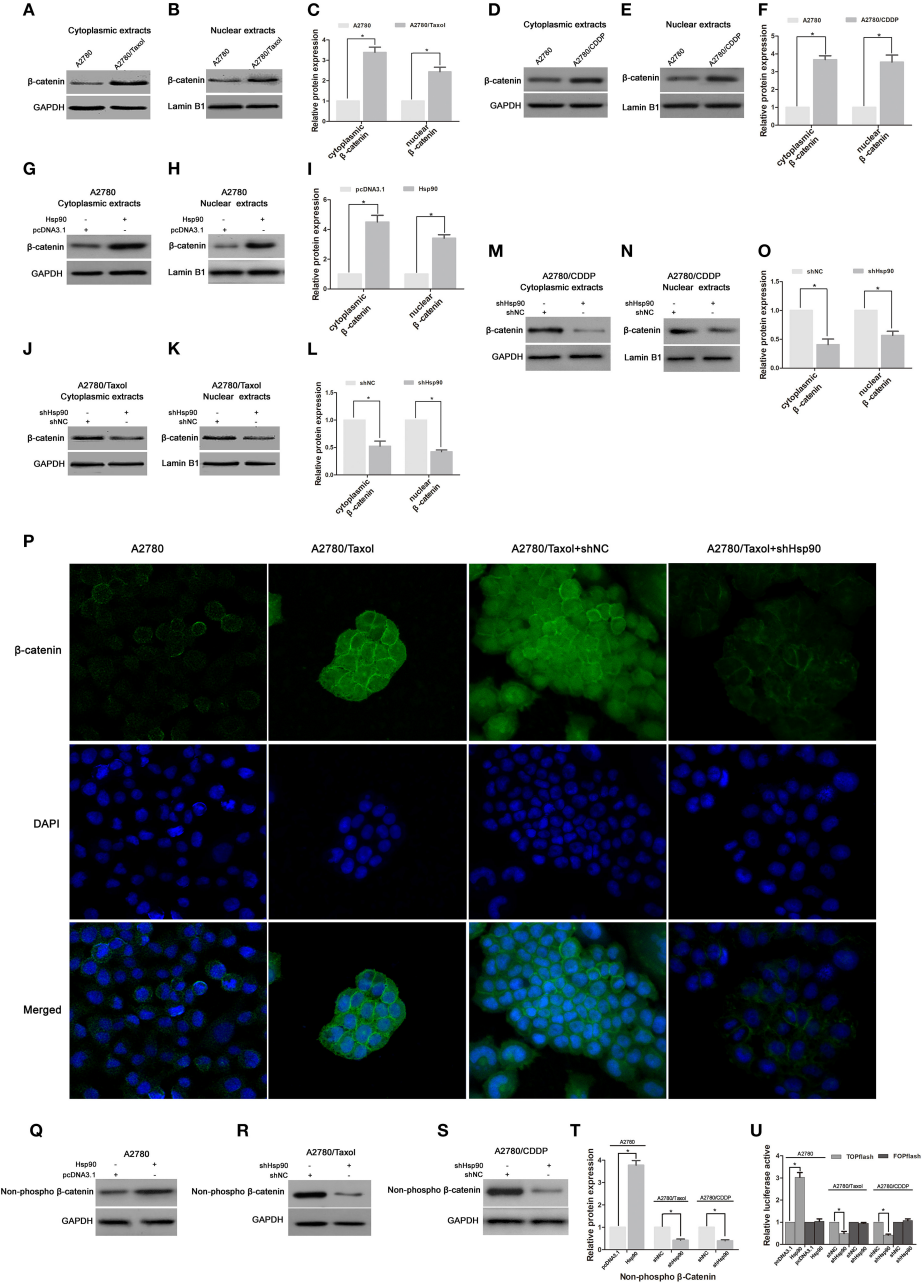
The effect of heat shock protein 90 (Hsp90) on nuclear translocation and transcriptional activity of β-catenin. Respective western blots and relative quantitation for cytoplasmic and nuclear β-catenin in A2780, A2780/Taxol, and A2780/CDDP cells **(A–F)**, A2780 cells transfected with pcDNA3.1 or pcDNA3.1-Hsp90 for 72 h **(G–I)**, A2780/Taxol and A2780/CDDP cells transfected with shNC or shHsp90 for 72 h **(J–O)**. **(P)** The subcellular localization of β-catenin was analyzed using immunofluorescence staining in A2780, A2780/Taxol, and A2780/Taxol cells transfected with shNC or shHsp90 for 72 h. Green, β-catenin; blue, nuclear DNA. Respective western blots and relative quantitation for non-phospho (active) β-catenin in A2780 cells transfected with pcDNA3.1 or pcDNA3.1-Hsp90 for 72 h, A2780/Taxol and A2780/CDDP cells transfected with shNC or shHsp90 for 72 h cells **(Q–T)**. **(U)** Dual-luciferase reporter assay for TOPflash and FOPflash luciferase activity in A2780 cells transfected with pcDNA3.1 or pcDNA3.1-Hsp90 for 48 h, A2780/Taxol and A2780/CDDP cells transfected with shNC or shHsp90 for 48 h. The relative luciferase activity was normalized against Renilla reporter pRL-SV40 activity. The results of western blotting were analyzed using ImageJ (mean ± SD of 3 independent experiments). *P < 0.05.

It is well-established that the nuclear translocation of β-catenin is required for its target gene expression (46). To further understand the mechanism by which Hsp90 promoted the expression of P-gp, BCRP, Survivin and Bcl-2 via regulating β-catenin, the effect of Hsp90 on subcellular localization of β-catenin was examined in A2780 and A2780/Taxol cells. Western blotting showed that the protein levels of both cytoplasmic and nuclear β-catenin were increased in A2780/Taxol and A2780/CDDP cells compared with the levels in A2780 cells (Figures 6A–F), and upregulated in Hsp90 overexpressing A2780 cells (Figures 6G–I), but decreased in Hsp90 silencing A2780/Taxol and A2780/CDDP cells (Figures 6J–O). The above results were further confirmed by immunofluorescence microscopy analysis (Figure 6P). These data suggested that Hsp90 facilitated the cytoplasmic accumulation and nuclear translocation of β-catenin.

Moreover, the protein level of non-phospho (active) β-catenin was increased in Hsp90 overexpressing A2780 cells, while reduced by shHsp90 in A2780/Taxol and A2780/CDDP cells (Figures 6Q–T). We further examined whether Hsp90 regulated the transcriptional activity of β-catenin. TOPflash and FOPflash luciferase reporters, which, respectively, include wildtype and mutant β-catenin/TCF-binding site, are widely used to characterize β-catenin transcriptional activity in nucleus (44). Dual-luciferase reporter assay showed that TOPflash luciferase activity of A2780 cells transfected with recombinant vector of Hsp90 was increased relative to the cells transfected with pcDNA3.1. Meanwhile, TOPflash luciferase activities of A2780/Taxol and A2780/CDDP cells transfected with shHsp90 were reduced relative to the cells transfected with shNC. However, there was no significant difference in FOPflash luciferase activity (Figure 6U). Above data suggested that Hsp90 enhanced transcriptional activity of β-catenin.

### AKT/GSK3β/β-Catenin Signaling Was Essential for the Hsp90-Mediated MDR of Ovarian Cancer

**Figure 7:**
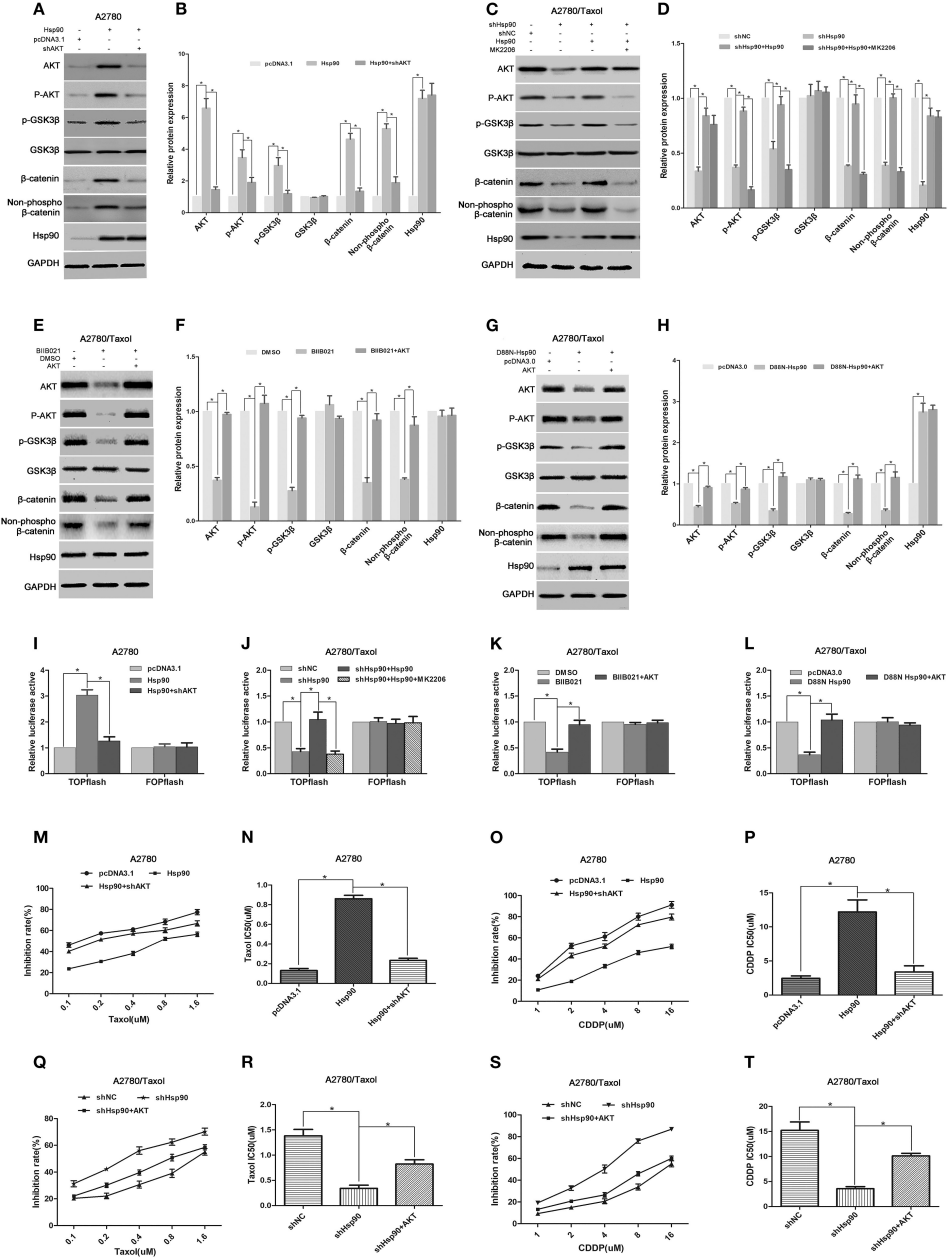
The role of AKT/GSK3β/β-catenin signaling in Hsp90-induced multi-drug resistance (MDR). Respective western blots and relative quantitation for AKT, p-AKT, p-GSK3β, GSK3β, total β-catenin, non-phospho (active) β-catenin, and Hsp90 in A2780 cells transfected with pcDNA3.1, pcDNA3.1-Hsp90, or shAKT plus pcDNA3.1-Hsp90 for 72 h **(A,B)**, A2780/Taxol cells transfected with shNC, shHsp90, or pcDNA3.1-Hsp90 plus shHsp90 for 72 h, and then treated with or without MK2206 for 6 h **(C,D)**, A2780/Taxol cells incubated with DMSO, BIIB021, or BIIB021 plus transfection with pcDNA3.0-AKT for 72 h **(E,F)**, and A2780/Taxol cells transfected with pcDNA3.0, pcDNA3.0-D88N-Hsp90, or pcDNA3.0-D88N-Hsp90 plus pcDNA3.0-AKT for 72 h **(G,H)**. Dual-luciferase reporter assay for TOPflash and FOPflash luciferase activity in A2780 cells transfected with pcDNA3.1, pcDNA3.1-Hsp90, or shAKT plus pcDNA3.1-Hsp90 for 48 h **(I)**, A2780/Taxol cells transfected with shNC, shHsp90, or pcDNA3.1-Hsp90 plus shHsp90 for 48 h, and then treated with or without MK2206 for 6 h **(J)**, A2780/Taxol cells incubated with DMSO, BIIB021, or BIIB021 plus transfection with pcDNA3.0-AKT for 48 h **(K)**, and A2780/Taxol cells transfected with pcDNA3.0, pcDNA3.0-D88N-Hsp90, or pcDNA3.0-D88N-Hsp90 plus pcDNA3.0-AKT for 48 h **(L)**. The sensitivity and IC50 of paclitaxel **(M,N)** or cisplatin **(O,P)** in A2780 cells transfected with pcDNA3.1, pcDNA3.1-Hsp90, or pcDNA3.1-Hsp90 plus shAKT for 72 h. The sensitivity and IC50 of paclitaxel **(Q,R)** or cisplatin **(S,T)** in A2780/Taxol cells transfected with shNC, shHsp90, or shHsp90 plus pcDNA3.0-AKT for 72 h. The results of western blotting were analyzed using ImageJ (mean ± SD of 3 independent experiments). *P < 0.05.

To further understand the underlying mechanism by which Hsp90 regulated β-catenin, we determined the effect of Hsp90 on AKT/GSK3β/β-catenin signaling. Western blotting results showed that the protein levels of AKT, P-AKT (Ser473), p-GSK3β (Ser9), total β-catenin, and non-phospho (active) β-catenin were increased following Hsp90 overexpression in A2780 cells, while the Hsp90-induced expression of these proteins were abolished by the shRNA-mediated knockdown of AKT (Figures 7A,B). Moreover, the expressions of AKT, P-AKT (Ser473), p-GSK3β (Ser9), total β-catenin, and non-phospho (active) β-catenin were reduced by silencing of Hsp90 in A2780/Taxol cells, and the overexpression of Hsp90 reversed such a decline. However, the AKT inhibitor MK2206 eliminated the effect of Hsp90 overexpression on P-AKT (Ser473), p-GSK3β (Ser9), total β-catenin, and non-phospho (active) β-catenin levels (Figures 7C,D). These results suggested that Hsp90 increased β-catenin accumulation via activating AKT/GSK3β signaling. To confirm our observation, A2780/Taxol cells were incubated with BIIB021 or transfected with D88N-Hsp90, in the presence or absence of AKT overexpression. The results showed that AKT, P-AKT (Ser473), p-GSK3β (Ser9), total β-catenin, and non-phospho (active) β-catenin levels were downregulated by BIIB021 or D88N-Hsp90, and this reduction was rescued by the ectopic expression of AKT (Figures 7E–H). Furthermore, the transcriptional activity of β-catenin was assayed using TOPflash and FOPflash luciferase reporters. Dual-luciferase reporter assay showed that TOPflash luciferase activity was increased by Hsp90 overexpression, while reduced by shHsp90, BIIBO21 or D88N-Hsp90. Hsp90-increased TOPflash activity was inhibited by shAKT or MK2206, while shHsp90-, BIIBO21-, or D88N-Hsp90-decreased TOPflash activity was rescued by AKT overexpression (Figures 7I–L). However, no significant difference was observed in FOPflash luciferase activity. Above results confirmed that Hsp90 promoted AKT/GSK3β/β-catenin signaling.

Next, we explored the role of AKT in the Hsp90-induced MDR of ovarian cancer. The IC50 values in ovarian cancer cells incubated with paclitaxel or cisplatin were determined by the MTT assay. The results showed that the sensitivity of A2780 cells to paclitaxel (IC50 0.13 μM vs. 0.86 μM) or cisplatin (IC50 2.46 μM vs. 12.19 μM) was impaired by the overexpression of Hsp90, whereas Hsp90-decreased sensitivity was restored by the knockdown of AKT (paclitaxel IC50 0.86 μM vs. 0.23 μM; cisplatin IC50 12.19 μM vs. 3.38 μM) (Figures 7M–P). Moreover, our preliminary data showed that the resistance of A2780/Taxol cells to paclitaxel (IC50 1.38 μM vs. 0.34 μM) or cisplatin (IC50 15.22 μM vs. 3.56 μM) was reduced by Hsp90 silencing, while this reduction was rescued by the ectopic expression of AKT (paclitaxel IC50 0.34 μM vs. 0.82 μM) or (cisplatin IC50 3.56 μM vs. 10.12 μM) (Figures 7Q–T). Collectively, the above results suggested that Hsp90 enhanced AKT/GSK3β/β-catenin signaling to drive MDR in ovarian cancer.

## Discussion

Surgical resection and chemotherapy are the main treatments for ovarian cancer (47). Unfortunately, chemotherapy often fails due to emergence of MDR (48, 49). Thus, elucidating the mechanism of MDR is key to the successful treatment of ovarian cancer. Hsp90 is a molecular chaperone with highly conserved structure that mediates cell proliferation, differentiation and apoptosis (50–52). Previous studies have shown that abnormal activation of Hsp90 contributes to the development and progression of various cancers (53–55). This study aimed to investigate the roles and mechanisms of Hsp90 in the MDR of ovarian cancer.

To investigate the correlation between the MDR of ovarian cancer and Hsp90, we first compared Hsp90 expression between drug-resistant cells (A2780/Taxol and A2780/CDDP) and their parental cells (A2780). It was found that Hsp90 was overexpressed in A2780/Taxol and A2780/CDDP cells (Figures 1A,B). To examine the role of Hsp90 in the regulation of paclitaxel and cisplatin resistance in ovarian cancer, the expression of Hsp90 was silenced using shRNA. Although A2780/Taxol and A2780/CDDP cells, respectively, exhibited much stronger resistance to paclitaxel and cisplatin compared with A2780 cells, silencing Hsp90 increased the sensitivity of A2780, A2780/Taxol and A2780/CDDP cells to paclitaxel or cisplatin, and decreased the IC50 value (Figures 1C–J). BIIB021 is a fully synthetic small-molecule inhibitor of Hsp90, that binds to the ATP-binding pocket of Hsp90 and interferes with its chaperone function (56). To further clarify the specific function of Hsp90 in drug-resistance in ovarian cancer, Hsp90 was inhibited using BIIB021. Similar with above observations, BIIB021 remarkably enhanced the chemosensitivity of ovarian cancer cells, and significantly reduced the IC50 (Figures 1K–N). These data suggested that Hsp90 contributed to drug-resistance in ovarian cancer cells. Next, we focused on the role of Hsp90 in the MDR of ovarian cancer. Cancer cells that acquire resistance to 1 drug are generally also resistant to other structurally and functionally unrelated chemotherapeutic agents, a phenomenon defined as MDR (57). Once MDR is acquired, the therapeutic effects of anti-cancer drugs are impaired (58). Interestingly, we found that A2780/Taxol cells were cross-resistant to cisplatin, and A2780/CDDP cells were also endowed with the ability to resist paclitaxel, suggesting that they can be used as an MDR cell model. Suppressing Hsp90 using shRNA or BIIB021 also alleviated the cross-resistance of A2780/Taxol and A2780/CDDP cells to cisplatin and paclitaxel, and markedly reduced the IC50 values (Figures 1O–V). This further demonstrated that Hsp90 contributed to the MDR of ovarian cancer and that the downregulation of Hsp90 chemosensitized drugs-induced cytotoxicitises in multi-drug resistant ovarian cancer cells. However, the mechanism by which Hsp90 mediates the MDR of ovarian cancer remains unclear.

The potential mechanisms of MDR, which can be either intrinsic or acquired after chemotherapy, have been reported, including ATP-driven drug efflux from the cell, apoptosis evasion, autophagy induction and cancer stem cell regulation (59–61). Here, we found that ABC transporters including P-gp and BCRP, which can recognize and actively extrude various cytotoxic drugs from cells (62), were overexpressed in multi-drug resistant ovarian cancer cells (Figures 2A,B), indicating that P-gp and BCRP were involved in the MDR of ovarian cancer. Moreover, the expression levels of P-gp and BCRP were increased by the ectopic expression of Hsp90 in A2780 cells (Figures 2C,D) and IOSE-80 cells (Supplementary Figure 1), but reduced by the downregulation of Hsp90 using shHsp90 or BIIB021 in A2780/Taxol cells (Figures 2E–H). Furthermore, Hsp90-induced resistance to paclitaxel was impaired by the inhibitor of P-gp or BCRP (Figures 2I,J). However, Hsp90-induced resistance to cisplatin was not significantly affect by these inhibitors (Figures 2K,L), this may be attributed to the fact that cisplatin is not a substrate of P-gp and BCRP (63). Above results suggested that Hsp90 trigger chemoresistance via P-gp and BCRP in ovarian cancer, inhibiting Hsp90 could improve the drug sensitivity. Notably, previous study has hinted a risk that some Hsp90 inhibitor as anticancer agent could be themselves substrates of ABC transporters (64). The resistance to two Hsp90 inhibitors (benzoquinone ansamycins GdA and herbimycin A) was observed in drug-resistant cancer cells overexpressing P-gp (65). Another Hsp90 inhibitor 17-AAG was also found to be less effective in cells overexpressing ABC transporters (66–68). Fortunately, the synthetic purine- and pyrazole-based inhibitors of Hsp90 such as BIIB021 used in this study, which are not P-gp substrate, can evade ABC transporters-mediated MDR mechanism in cancer cells (69). Therefore, it is necessary to insure that the inhibitor designed to target HSP90 for reversing drug-resistance, should be “poor substrates” of ABC transporters.

The acquisition of an MDR phenotype is not limited to the ectopic expression of ABC transporters. For example, anti-apoptotic proteins also facilitate the development of MDR (70, 71). Survivin is an important member of the apoptosis inhibitor protein family, that is frequently found to be upregulated in various malignancies and associated with poor prognosis and drug resistance of cancers (72). Similarly, Bcl-2 is also an anti-apoptotic protein that inhibits programmed cell death (73) and is believed to play an important role in cell survival and drug resistance of lymphomas, colorectal cancer, prostate cancer, and other malignancies (74). In this study, both Survivin and Bcl-2 were significantly overexpressed in multi-drug resistant ovarian cancer cells (Figures 3A,B). Moreover, Hsp90 positively regulated Survivin and Bcl-2 expression in A2780 and A2780/Taxol cells (Figures 3C–H). Interestingly, there is no significant difference in apoptosis between the parental and the drug-resistant ovarian cancer cells, in the absence of anticancer drug stimulus. Although the pretreatment of paclitaxel or cisplatin significantly increased apoptosis of A2780 cells, A2780/Taxol and A2780/CDDP cells were cross-resistant to paclitaxel- and cisplatin-induced apoptosis. Fortunately, downregulation of Hsp90 using shRNA or BIIB021 can significantly augment paclitaxel- and cisplatin-induced apoptosis in different ovarian cancer cells mentioned above (Figure 4 and Supplementary Figure 3). Recently, the sustained high expression of Survivin and Bcl-2 is shown to protect cancer cells from drug-induced apoptosis, thereby driving MDR (75–78). Inhibiting Survivin or Bcl-2 can improve chemosensitivity of cancer cells to paclitaxel and cisplatin (79, 80). Therefore, the above data suggested that Hsp90 induced MDR of ovarian cancer cells to paclitaxel and cisplatin by regulating Survivin and Bcl-2. Taken together, our results suggested that Hsp90 enhanced the expression of ABC transporters in favor of drug efflux and upregulated anti-apoptosis proteins against drug-induced apoptosis, resulting in the MDR of ovarian cancer. Interestingly, recent study suggested that the mechanism of HSP90-mediated resistance could be not only limited to above pathway, but also be involved in the upregulating DNA repair pathways. This is confirmed by the results revealing that Hsp90 inhibitor could cause suppression of DNA repair mechanisms to enhance drug-mediated DNA damage in cisplatin-resistant cancer cells, and finally reversed the resistance phenotype (81).

We next explored the mechanism involved in the Hsp90-mediated expression of P-gp, BCRP, Survivin and Bcl-2. Previous studies have confirmed that P-gp, BCRP, Survivin and Bcl-2 are target genes of β-catenin (25, 27, 28, 30). In addition, the role of β-catenin regulating chemoresistance in multiple cancers was previously reported (82, 83). Therefore, we determined the role of β-catenin in the Hsp90-mediated expression of ABC transporters and anti-apoptosis proteins in ovarian cancer. The accumulation of β-catenin in cells is one important step to exert its function (84). Our results revealed that β-catenin was overexpressed in A2780/Taxol cells compared with A2780 cells, and the upregulation of β-catenin was accompanied by the overexpression of Hsp90 in A2780 cells (Figures 5A–D). Moreover, the protein level of β-catenin was decreased by silencing or inhibiting of Hsp90 using shRNA or BIIB021, respectively, while shHsp90-decreased β-catenin expression was rescued by the overexpression of Hsp90 (Figures 5E–H). These results suggested that Hsp90 enhanced the accumulation of β-catenin. To further confirm this result, D88N-Hsp90, a dominant-negative construct that blocks the ATP-binding site and molecular chaperone function of Hsp90 (43), was used to suppress Hsp90 activity. The results showed that the protein level of β-catenin was reduced by D88N-Hsp90 (Supplementary Figure 2). Therefore, we hypothesized that Hsp90 increased β-catenin accumulation to regulate the expression of P-gp, BCRP, Survivin and Bcl-2. Our hypothesis was confirmed by the results showing that silencing β-catenin abolished the upregulation of P-gp, BCRP, Survivin and Bcl-2 mediated by Hsp90 (Figures 5I–L). It is well-known that the nuclear translocation of β-catenin following its accumulation is an essential step to trigger downstream target gene expression (85). Here, we further assessed whether altered Hsp90 protein levels affected the nuclear translocation of β-catenin. Western blotting showed that cytoplasmic and nuclear β-catenin expressions were positively controlled by Hsp90 (Figures 6A–L). Consistent results were obtained by immunofluorescence staining (Figure 6M). In addition, Hsp90 positively regulated the protein level of active β-catenin and the transcriptional activity (Figures 6N–R). Collectively, the above results suggested that Hsp90 enhanced the accumulation, nuclear translocation and transcriptional activity of β-catenin to increase expressions of P-gp, BCRP, Survivin, and Bcl-2.

Growing evidence has revealed that β-catenin accumulation is increased after an event driven by the AKT-mediated inactivation of GSK3β (46, 86, 87). Activated AKT phosphorylates GSK3β at Ser9/21 to inhibit GSK3β (88). The inactivated GSK3β prevents β-catenin from phosphorylation and ubiquitination degradation, resulting in its cytoplasmic accumulation and nuclear translocation of β-catenin (85). To understand the mechanism by which Hsp90 regulated β-catenin in ovarian cancer cells, we examined the role of AKT/GSK3β signaling in Hsp90-mediated β-catenin expression. The results showed that the expression levels of AKT, P-AKT (Ser473), and P-GSK3β (Ser9) were increased by the overexpression of Hsp90 (Figures 7A,B), and decreased by the downregulation of Hsp90 using shRNA, BIIB021 or D88N-Hsp90 (Figures 7C–H). The Hsp90-induced expression of AKT, P-AKT (Ser473), and P-GSK3β (Ser9) were eliminated by shAKT or MK2206, while their decreased expression following Hsp90 downregulation were reversed by the ectopic expression of AKT (Figures 7E–H). These results suggested that Hsp90 promoted AKT expression and enhanced AKT/GSK3β signaling. Hsp90 can directly interact with and protect AKT against degradation, which may explain the Hsp90-mediated increase in AKT expression (89). Moreover, the Hsp90-induced expressions of total and active β-catenin were abolished by shAKT or MK2206 (Figures 7A–D), whereas the BIIB021- or D88N-Hsp90-inhibited expressions of total and active β-catenin were rescued by the ectopic expression of AKT (Figures 7E–H). Dual-luciferase reporter assay further confirmed that AKT was responsible for Hsp90-induced transcriptional activation of β-catenin (Figures 7I–L). Collectively, the above results suggested that Hsp90 promoted accumulation and transcriptional activity of β-catenin by enhancing AKT/GSK3β signaling, in other words, Hsp90 enhanced AKT/GSK3β/β-catenin signaling. Furthermore, our results showed that Hsp90-induced resistance to paclitaxel and cisplatin in A2780 cells was impaired by the knockdown of AKT (Figures 7M–P), whereas shHsp90-reduced resistance to paclitaxel and cisplatin in A2780/Taxol cells was restored by the ectopic expression of AKT (Figures 7Q–T). Together, the above results suggested that AKT/GSK3β/β-catenin signaling was critical for the Hsp90-driven MDR of ovarian cancer.

In conclusion, we have presented evidence that Hsp90 triggers MDR in ovarian cancer. Specifically, Hsp90 enhanced AKT/GSK3β/β-catenin signaling to upregulate ABC transporters and anti-apoptosis proteins closely associated with MDR. Suppressing Hsp90 re-sensitized multi-drug resistant ovarian cancer cells via downregulating AKT/GSK3β/β-catenin signaling. This study conclusively indicates that targeting Hsp90 is a promising therapeutic strategy for ovarian cancer patients who have developed MDR.

## Data Availability Statement

The original contributions presented in the study are included in the article/Supplementary Material, further inquiries can be directed to the corresponding author/s.

## Author Contributions

KZ and YZ: conception, design of research, edited, revised, and approved final version of manuscript. LY, YY, WZ, YX, ZH, HH, and LP: performed experiments. LY, YY, and WZ: analyzed data, prepared figures, and drafted manuscript. LY, YY, WZ, KZ, and YZ: interpreted results of experiments. All authors contributed to the article and approved the submitted version.

## Conflict of Interest

The authors declare that the research was conducted in the absence of any commercial or financial relationships that could be construed as a potential conflict of interest.

## Supplementary Material

Supplementary Figure 1 Overexpression of Hsp90 upregulated P-gp and BCRP in IOSE80 cells. Western blots **(A)** and relative quantitation **(B)** of P-gp, BCRP and Hsp90 in human normal ovarian surface epithelia cells IOSE80 transfected with pcDNA3.1-Hsp90 for 72 h. GAPDH was used as the internal control. The results of western blotting were analyzed using ImageJ (mean ± SD of 3 independent experiments). ^*^*P* < 0.05.

Supplementary Figure 2 D88N-Hsp90 decreased the expression of β-catenin. Western blots **(A)** and relative quantitation **(B)** of β-catenin and Hsp90 in A2780/Taxol cells transfected with pcDNA3.0-D88N-Hsp90 (a dominant negative gene of Hsp90) for 72 h. GAPDH was used as the internal control. The results of western blotting were analyzed using ImageJ (mean ± SD of 3 independent experiments). ^*^*P* < 0.05.

Supplementary Figure 3 Inhibition of Hsp90 promoted cisplatin- and paclitaxel- induced apoptosis of A2780/CDDP cells. Flow cytometry analysis and apoptotic rates in A2780/CDDP cells transfected with shNC or shHsp90 for 24 h, or incubated with 1 μM BIIB021 for 24 h, followed by treatment with 2 μM cisplatin **(A–F)** or 0.4 μM paclitaxel for 48 h **(G–L)**, as indicated. Data are expressed as mean ± SD of 3 for each experimental group. ^*^*P* < 0.05.
