# Supplementary figures and images for: Heat Shock Protein 90 Triggers Multi-Drug Resistance of Ovarian Cancer via AKT/GSK3β/β-Catenin Signaling
Source: Front Oncol. 2021 Mar 2;11:620907. doi: 10.3389/fonc.2021.620907 (PMC7960917; doi:10.3389/fonc.2021.620907)

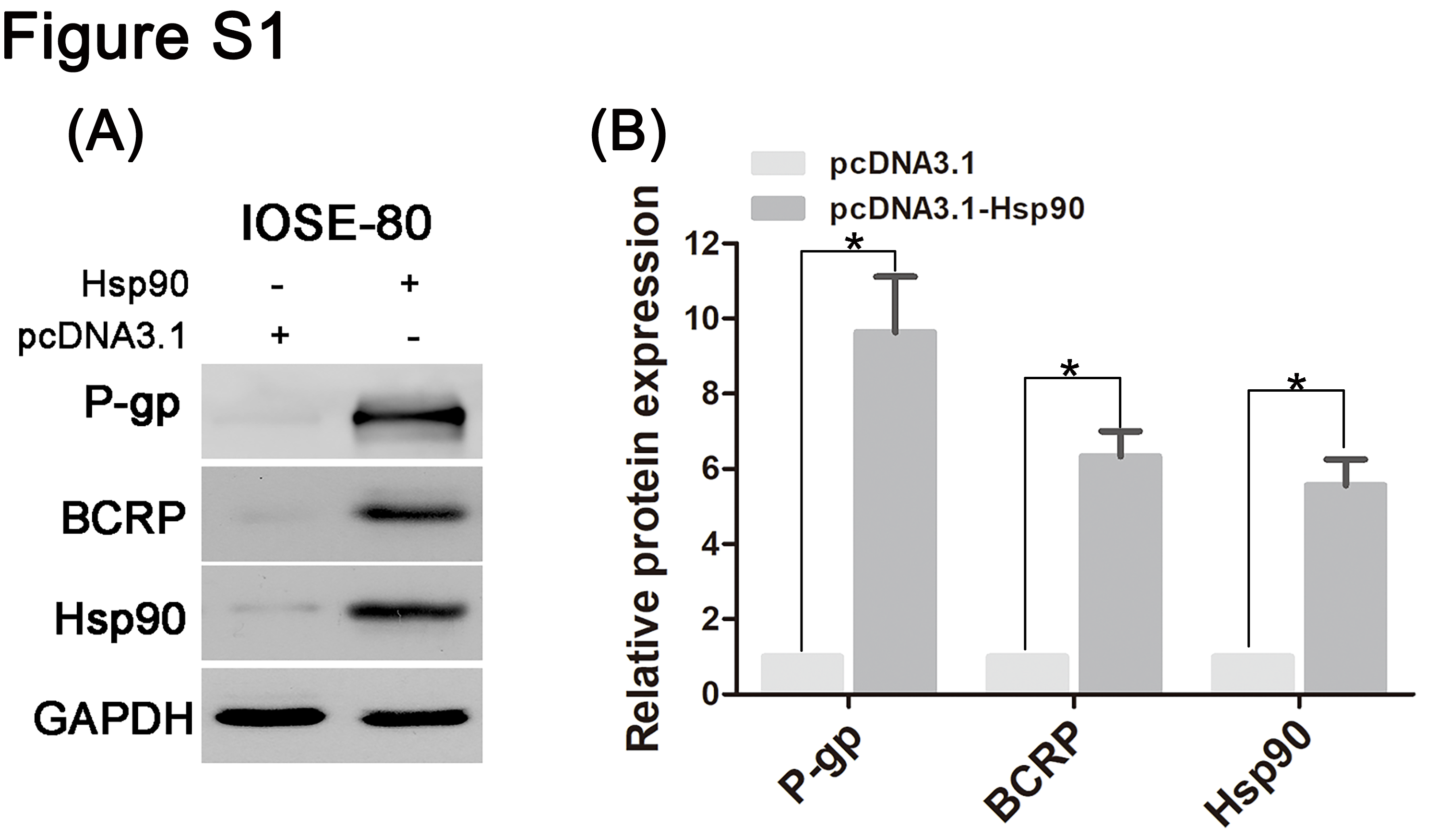

Supplement: Supplementary Figure 1 — Overexpression of Hsp90 upregulated P-gp and BCRP in IOSE80 cells. Western blots (A) and relative quantitation (B) of P-gp, BCRP and Hsp90 in human normal ovarian surface epithelia cells IOSE80 transfected with pcDNA3.1-Hsp90 for 72 h. GAPDH was used as the internal control. The results of western blotting were analyzed using ImageJ (mean ± SD of 3 independent experiments). *P < 0.05. [file Image_1.TIF]

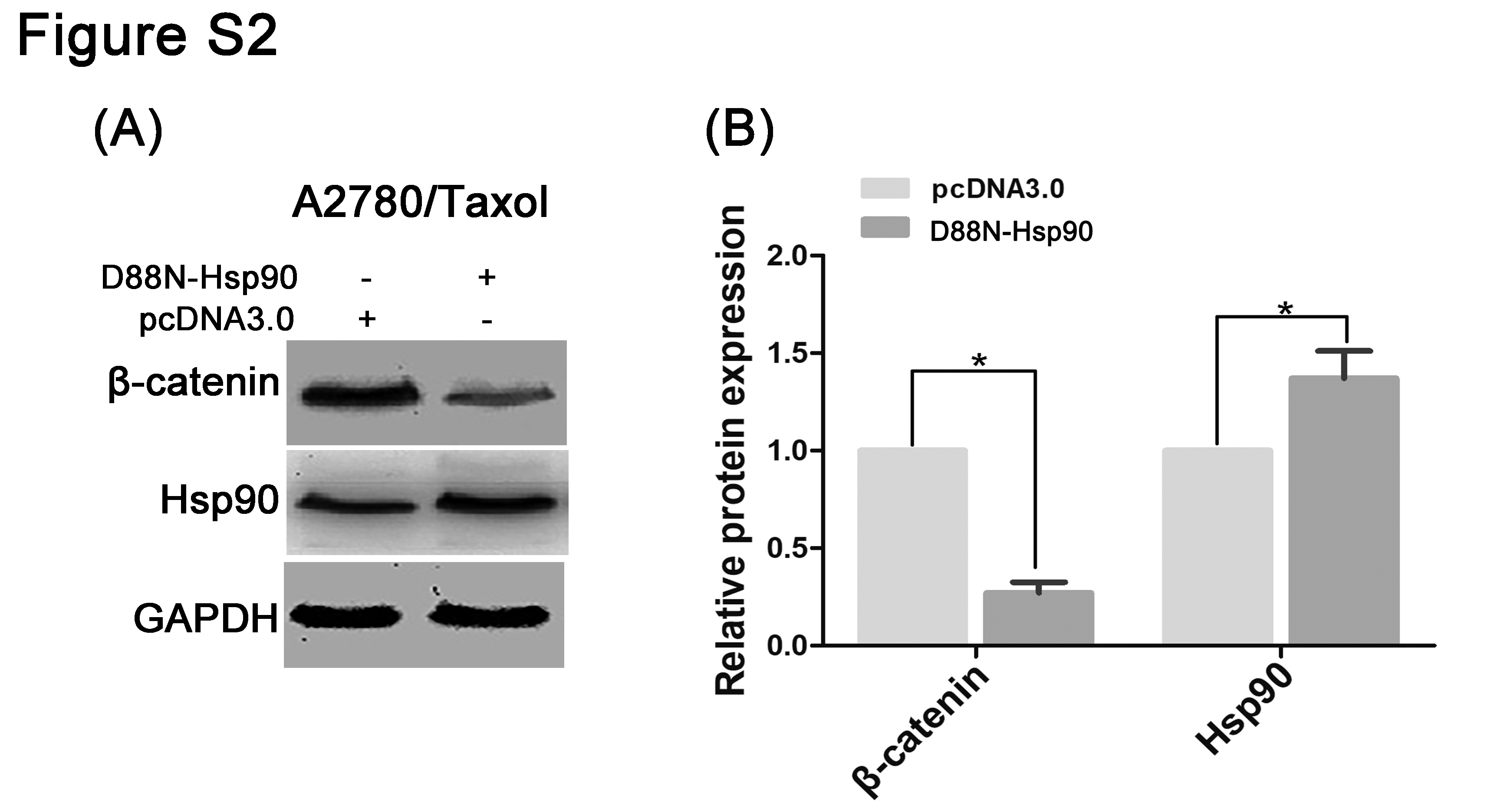

Supplement: Supplementary Figure 2 — D88N-Hsp90 decreased the expression of β-catenin. Western blots (A) and relative quantitation (B) of β-catenin and Hsp90 in A2780/Taxol cells transfected with pcDNA3.0-D88N-Hsp90 (a dominant negative gene of Hsp90) for 72 h. GAPDH was used as the internal control. The results of western blotting were analyzed using ImageJ (mean ± SD of 3 independent experiments). *P < 0.05. [file Image_2.TIF]

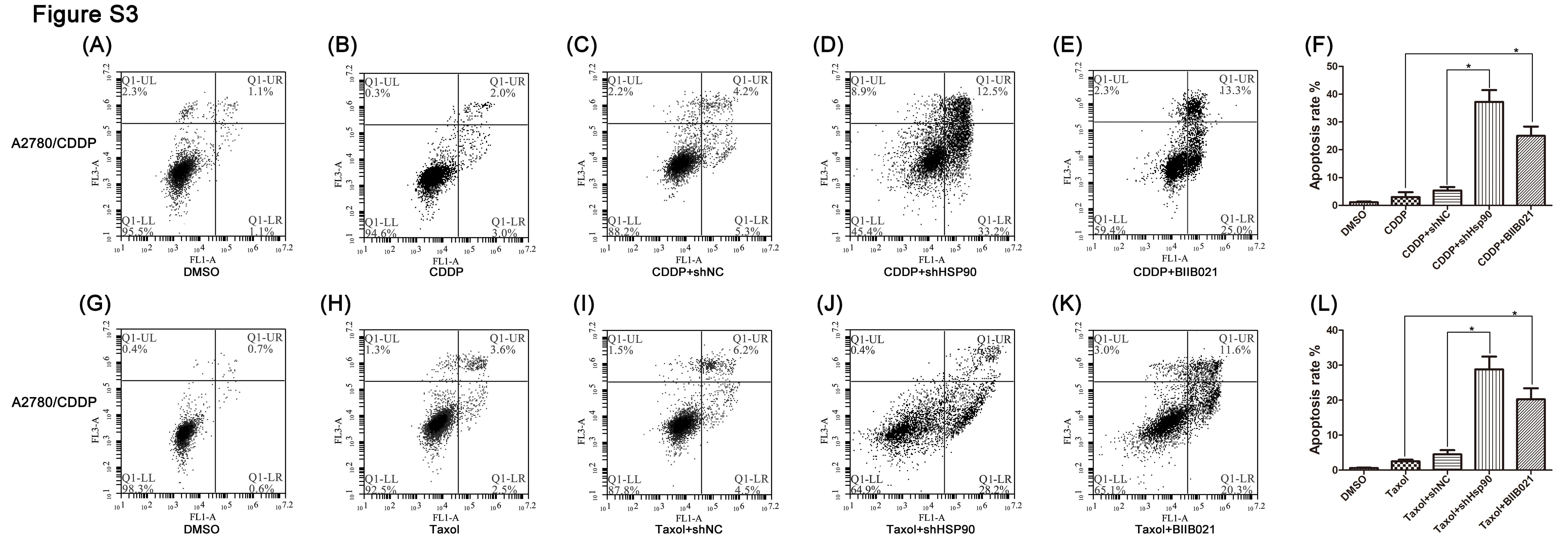

Supplement: Supplementary Figure 3 — Inhibition of Hsp90 promoted cisplatin- and paclitaxel- induced apoptosis of A2780/CDDP cells. Flow cytometry analysis and apoptotic rates in A2780/CDDP cells transfected with shNC or shHsp90 for 24 h, or incubated with 1 μM BIIB021 for 24 h, followed by treatment with 2 μM cisplatin (A–F) or 0.4 μM paclitaxel for 48 h (G–L), as indicated. Data are expressed as mean ± SD of 3 for each experimental group. *P < 0.05. [file Image_3.TIF]
